# Identification and *in vitro* functional assessment of 10 CYP2C9 variants found in Chinese Han subjects

**DOI:** 10.3389/fendo.2023.1139805

**Published:** 2023-03-15

**Authors:** Qing Zhang, Yuying Qi, Shuanghu Wang, Fangling Zhao, Lili Zou, Quan Zhou, Peiwu Geng, Yun Hong, Hang Yang, Qingfeng Luo, Jianping Cai, Hualan Wu, Dongxu Wang, Hao Chen, Jiefu Yang, Dapeng Dai

**Affiliations:** ^1^ Department of Cardiovascular, Beijing Hospital, National Center of Gerontology, Beijing, China; ^2^ Institute of Geriatric Medicine, Chinese Academy of Medical Sciences, Beijing, China; ^3^ Graduate School of Peking Union Medical College, Chinese Academy of Medical Sciences, Beijing, China; ^4^ The Key Laboratory of Geriatrics, Beijing Institute of Geriatrics, Institute of Geriatric Medicine, Chinese Academy of Medical Sciences, Beijing Hospital/National Center of Gerontology of National Health Commission, Beijing, China; ^5^ Beijing Institute of Geriatrics, Peking University Fifth School of Clinical Medicine, Beijing, China; ^6^ Laboratory of Clinical Pharmacy, The Sixth Affiliated Hospital of Wenzhou Medical University, The People’s Hospital of Lishui, Lishui, China; ^7^ Department of Gastroenterology, Beijing Hospital, National Center of Gerontology, Beijing, China

**Keywords:** CYP2C9, allelic variant, genetic polymorphism, drug metabolism, Chinese Han population

## Abstract

Cytochrome P450 2C9 (CYP2C9) participates in about 15% of clinical drug metabolism, and its polymorphism is associated with individual drug metabolism differences, which may lead to the adverse drug reactions (ADRs). In this study, 1163 Chinese Han individuals were recruited to investigate their distribution pattern of *CYP2C9* gene and find out the variants that may affect their drug metabolic activities. We successfully developed a multiplex PCR amplicon sequencing method and used it for the genetic screening of *CYP2C9* in a large scale. Besides the wild type CYP2C9*1, totally 26 allelic variants of *CYP2C9* were detected, which included 16 previously reported alleles and 10 new non-synonymous variants that had not been listed on the PharmVar website. The characteristics of these newly detected CYP2C9 variants were then evaluated after co-expressing them with CYPOR in *S. cerevisiae* microsomes. Immunoblot analysis revealed that except for Pro163Ser, Glu326Lys, Gly431Arg and Ile488Phe, most of newly detected variants showed comparable protein expression levels to wild type in yeast cells. Two typical CYP2C9 probe drugs, losartan and glimepiride, were then used for the evaluation of metabolic activities of variants. As a result, 3 variants Thr301Met, Glu326Lys, and Gly431Arg almost lost their catalytic activities and most of other variants exhibited significantly elevated activities for drug metabolism. Our data not only enriches the knowledge of naturally occurring CYP2C9 variants in the Chinese Han population, but also provides the fundamental evidence for its potential clinical usage for personalized medicine in the clinic.

## 1 Introduction

Cytochrome P450 (CYP) is one of the critical enzymes involved in the drug metabolism in human. It is responsible for the biotransformation of most foreign substances, including 70-80% of clinically used drugs (1). Variation in clinical response to drug treatment is very common among individuals, and this variation can be affected by many factors, including age, gender, hormone, disease status, genetic polymorphism, and so forth (2–4). It is reported that most CYP enzymes exhibit marked genetic polymorphism, including copy number variation, missense mutation, insertion, deletion, and most of these genetic variations can affect the protein expression level or drug metabolic activity of enzyme. Clinical evidence has confirmed the apparent correlation between genetic polymorphisms of CYP and adverse drug reactions (ADRs), especially for drugs with narrow therapeutic windows (5–7).

The cytochrome P450 2C (CYP2C) subfamily is one of the most important members of the P450 family, with strong correlations with DNA and protein sequences (>82%) (1). Among them, CYP2C9 is the most abundantly expressed in human body, accounting for about 20% of the total liver P450 protein (8). CYP2C9 enzyme is responsible for the metabolism of approximately 15% of drugs, such as the hypoglycemic agent glimepiride and tolbutamide, the anticoagulant warfarin, the antihypertensive drug losartan, the anticonvulsant phenytoin, as well as the non-steroidal anti-inflammatory drugs flurbiprofen and diclofenac (9). Similar to other CYP2C members, the distribution pattern of *CYP2C9* polymorphic alleles varies greatly among different populations, and most of allelic variants exhibited significantly changed drug metabolic activities compared with that of the wild type CYP2C9 protein (10–12). In order to carry out the individualized treatment for patients with different pharmacogenetic phenotypes and reduce the occurrence rate of related ADRs, the Pharmacological Clinical Pharmacogenetics Implementation Consortium (CPIC) recently issued three CYP2C9-related guidelines for warfarin (13), phenytoin (14), and non-steroidal anti-inflammatory drugs, respectively (15).

To date, 85 allelic variants of *CYP2C9* gene have been discovered and nominated by the Pharmacogene Variation (PharmVar) Consortium (https://www.pharmvar.org/gene/CYP2C9, accessed on Dec 2022). Like other CYP2C members, *CYP2C9* gene is highly polymorphic and exhibits different distribution patterns in different races and geographical regions. According to the previous reports, CYP2C9*2 is the most prevalent defective allele in the Caucasian population (11.7%), while it is rarely identified in the Asian population (<0.1%) and African population (2.4%). In contrast, the main allelic variant of CYP2C9 in the Asian population is *3 (3.4% in East Asian and 11.3% in South Asian). CYP2C9*5, *6, *8, *9, and *11 are nearly only restricted to African populations, and CYP2C9*14 is almost uniquely found in South Asian individuals (16). Thus, clinical treatment decision on CYP2C9 meditated drugs in Caucasian populations may not have good generality and adaptability for other national populations. To better understand the specific polymorphic pattern of *CYP2C9* gene in the Chinese Han population, we previously conducted a large-scale genetic screening of *CYP2C9* in 2124 Chinese Han individuals and reported 21 new allelic variants in healthy subjects. Since then, four additional *CYP2C9* alleles CYP2C9*58-*60 and *62 were also identified in the warfarin-sensitive Chinese patients (17–20). Both *in vitro* and *in vivo* studies on these newly uncovered CYP2C9 variants revealed that almost all of them exhibited significantly changed metabolic activities, although their allele frequencies are below 1% (21–23). These data indicated that some other rare *CYP2C9* alleles may still be undiscovered and need further investigation, considering that more than 1.4 billion Chinese Han populations lived in mainland China.

In this study, 1163 healthy Chinese individuals were used for the genetic polymorphism investigation on *CYP2C9* gene by a time- and labor-saving sequencing method. As a consequence, 10 new allelic variants were identified and functional evaluation experiments were also conducted to characterize their impacts on the enzyme’s drug metabolic activity.

## 2 Materials and methods

### 2.1 Chemical materials

The FinePure Universal DNA Purification Kit was purchased from GENFINE Biotech (Beijing, China). The Taq plus master mix was obtained from Vazyme (Nanjing, China). PrimeSTAR Max DNA polymerase, restriction enzymes and DO Supplement-Ura were obtained from Takara Bio, Inc. (Otsu, Shiga, Japan). *Saccharomyces cerevisiae* strain YPH499 was obtained from ATCC (VA, USA). Yeast nitrogen base without amino acids, dextrose, galactose and losartan were purchased from Sigma-Aldrich (MO, USA). Baculosomes co-expressing human CYP2C9 and NADPH-cytochrome P450 oxidoreductase (OR) were purchased from BD Gentest (Woburn, MA, USA). The rabbit polyclonal anti-CYP2C9 antibody was obtained from Abcam (Cambridge, UK). The mouse monoclonal anti-OR antibody was from Santa Cruz Biotechnology (Dallas, Texas, USA). The Super Signal West Pico Trial Kit was obtained from Thermo Scientific (Rockford, IL, USA). Losartan was purchased from Sigma-Aldrich (St. Louis, MO, USA). Losartan carboxylic acid (E-3174), glimepiride and cyclohexyl hydroxymethyl glimepiride (M1) were obtained from Toronto Research Chemicals, Inc. (Toronto, Ontario, Canada). The NADPH-regenerating system was purchased from Promega (Madison, WI, USA). High-pressure liquid chromatography-grade solvents were purchased from Fisher Scientific Co. (Fair Lawn, NJ, USA). Other chemicals and solvents used were of analytical grade or the highest grade that was commercially available.

### 2.2 Genomic DNA extraction

All participants in this experiment were healthy Chinese Han individuals recruited in the Physical Examination Center of Beijing Hospital. The written informed consent form was signed when blood collection and this study was approved by the Ethics Committee of Beijing Hospital. FinePure Universal DNA Purification Kit was used to extract DNA from white blood cells following manufacturer’s recommend protocol, and genomic DNAs were diluted to the final concentration of approximately 40 ng/μL for PCR amplification.

### 2.3 Genotyping

**Figure 1:**
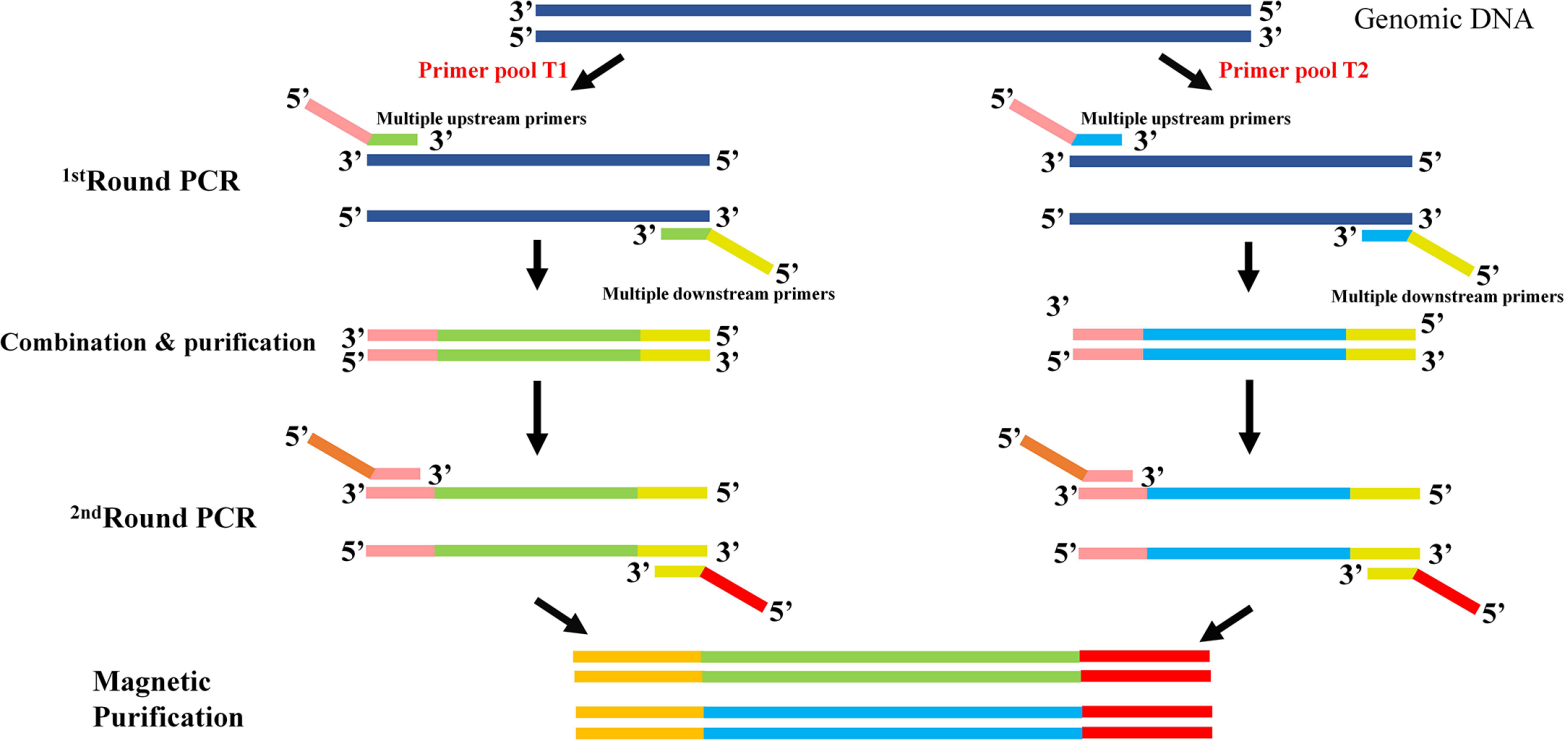
Schematic diagram of the multiplex PCR amplicon sequencing method for *CYP2C9* genotyping. Two rounds of PCR amplification were included in this method. The first round of PCR is for the multiplex amplification of all 9 exons of *CYP2C9* gene with pooled primers T1 or T2. After the combination and purification with magnetic beads, products were used as the template for the second round of PCR reaction and sequencing library construction. Universal primers in round 1 are illustrated as pink and yellow bars, and sequencing primers in round 2 are shown in brown and red bars.

To get a time-saving and cost-effective method for the genotyping of *CYP2C9* gene, a multiplex PCR amplicon sequencing method was developed in this study (**Figure 1**). The first round PCR reaction is used for the multiplex PCR amplification of all 9 exons of *CYP2C9* plus the exon-intron junction regions, and the second round of PCR reaction is aimed to obtain the amplicon library for the second-generation sequencing. Primers in the first round PCR reaction were designed by MFEPrimer (version 3.1) at the website of iGeneTech(https://mfeprimer3.igenetech.com/muld). Detailed primer information was listed in **Table 1**. A total amount of 40 ng genomic DNA was used as the input material for two rounds of PCR amplification. After purification with AMPure XP beads (Beckman, USA), barcoded library was quantified with Qubit 3.0 Fluorometer (Thermo Fisher Scientific, USA) and Agilent 2100 Bioanalyzer system (Agilent, USA) was used to measure the concentration and length of library fragments (from 270 to 420 bp). Qualified libraries were then sequenced on NovaSeq 6000 (Illumina, USA) with pair-end 150 sequencing strategy by iGeneTech Co (Beijing, China).

**Table 1:** Primers used for the first round of multiplex PCR reaction.

*Primer	**Forward (5’-3’)	**Reverse (5’-3’)	Amplicon (bp)	Position	Region
T1P1	TTGGAGTGCAAGCTCATGGTT	GTGAATTTACTTACCTTTTGCAAGCC	259	chr10:94938636-94938895	Exon 1
T1P2	GCAAGCTCATGGTTGTCTTAACAAG	AGGTGAATTTACTTACCTTTTGCAAGC	254	chr10:94938643-94938897	Exon 1
T1P3	GAGTGCAAGCTCATGGTTGTCT	GTGAATTTACTTACCTTTTGCAAGCCA	256	chr10:94938639-94938895	Exon 1
T1P4	ATTTGAAGCCTGTGTGGCTGA	ATGCAGCACCACTATGGGTTT	188	chr10:94941735-94941923	Exon 2
T2P1	GGACAAAATAGTAACTTCGTTTGCTGT	CATCCCCAAGACAGATGCTGAA	253	chr10:94941809-94942062	Exon 2
T1P5	TTCCTCTTTCTTGCCTGGGATC	GTAGTCCAGTAAGGTCAGTGATATGG	244	chr10:94942138-94942382	Exon 3
T2P2	GGGAGGATGGAAAACAGAGACT	CTTCCTCTTGAACACGGTCCT	243	chr10:94942062-94942305	Exon 3
T1P6	ACCCTGTGATCCCACTTTCATC	TGCACTTCAGAGCTTGATCCAT	250	chr10:94947782-94948032	Exon 4
T1P7	CCTGCAATGTGATCTGCTCCAT	TGCACTTCAGAGCTTGATCCATG	215	chr10:94947817-94948032	Exon 4
T2P3	AAACTACTATTATCTGTTAACAAATACAGTGTT	CAAAAATCTTGGCCTTACCTGGATC	259	chr10:94947698-94947957	Exon 4
T2P4	TCTGTTAACAAATACAGTGTTTTATATCTAAAGT	TCTCAGGAAGCAAAAATCTTGGC	257	chr10:94947710-94947967	Exon 4
T2P5	CTGTTAACAAATACAGTGTTTTATATCTAAAGTT	AGGAAGCAAAAATCTTGGCCTTAC	252	chr10:94947711-94947963	Exon 4
T1P8	GATCTGCTCCATTATTTTCCATAAACGT	GTCTGGGCAAGACTGTAGTATTCAA	233	chr10:94947827-94948060	Exon 4
T1P9	TCAATGGACATGAACAACCCTCA	GCTTCTCAAGCATTACTGATTGACC	173	chr10:94949224-94949397	Exon 5
T1P10	GGTTAGAATTGATCCTCTGGTCAGA	GTTGTGAGTTCCCGGGAAGTAA	260	chr10:94948898-94949158	Exon 5
T2P6	TGGTATATGGTATGTATGCTTTTATTAAAATCTT	GCTTTTGTTTACATTTTACCTTCTCCATT	260	chr10:94949043-94949303	Exon 5
T2P7	TTGGTATATGGTATGTATGCTTTTATTAAAATCT	CTTTTGTTTACATTTTACCTTCTCCATTTTCAT	260	chr10:94949042-94949302	Exon 5
T1P11	AGTTGGTCTACAGCCTCTGCTA	CTGTCCCAGCTCCAAACAAGT	243	chr10:94971939-94972182	Exon 6
T1P12	GCACAACCCTGAGATATGCTCT	ACCATGCCAGGCCAAGATATC	230	chr10:94972191-94972421	Exon 6
T2P8	TGCTGGTAAATAATTTGTCAGATAATTGCA	GACACTAGCAACACCTTCCCAA	252	chr10:94972058-94972310	Exon 6
T1P13	GCCATTTTTCTCCTTTTCCATCAGTTT	GTTGCAGTGTAGGAGAAACAAACTTAC	260	chr10:94981137-94981397	Exon 7
T1P14	TGTGCCATTTTTCTCCTTTTCCA	GAGAAACAAACTTACCTTGGGAATGAGA	251	chr10:94981134-94981385	Exon 7
T2P9	TCCAGGAAGAGATTGAACGTGTG	TTGGGGACTTCGAAAACATGGAG	231	chr10:94981188-94981419	Exon 7
T2P10	ATGCAAGACAGGAGCCACATG	GAGTTATGCACTTCTCTCACCCG	241	chr10:94981235-94981476	Exon 7
T2P11	CCAGGAAGAGATTGAACGTGTGA	TTGGGGACTTCGAAAACATGGA	230	chr10:94981189-94981419	Exon 7
T1P15	CCACTGTTTCTTCAACCTTCATGG	CTTGTACCCTGAAACACAAATGGA	246	chr10:94985968-94986214	Exon 8
T2P12	TGGTACTGCCCTTCTTTGGAAC	GTCAAACATCTCTGGGTTGGGA	205	chr10:94985902-94986107	Exon 8
T2P13	CTTCATGCCTTTCTCAGCAGGTA	TGGATTAACTCCCCAAAGTCCAC	222	chr10:94986154-94986376	Exon 8
T1P16	ACCCATCCACCCATCTATCTCT	GGTTCTTTGGGTCAACCAGAGA	255	chr10:94988699-94988954	Exon 9
T1P17	CCTGTCTGAAGAAGAGCAGATGG	CTCTCCGTAATGGAGGTCGAATG	170	chr10:94989019-94989189	Exon 9
T2P14	CATGAGGAGTAACTGCTCTCTGTG	GACTGCACAGCAGCAGC	255	chr10:94988808-94989063	Exon 9

*Primers starting with T1 and T2 were mixed in the primer pool T1 and T2, respectively.

**All primers contained a universal part in the 5’ direction and only the sequences for 3’ specific part were listed in this table.

Raw reads were filtered to remove low quality reads using FastQC (Version 0.11.9) and clean data were mapped to the reference genome GRCh38 and annotated using Annovar software (24). The high-quality annotated data were then obtained after filtering with these parameters: Sequencing depth >50 and detected frequency is within the range of 0.4-0.6 (heterozygote) or 0.9-1.0 (homozygote). Then, detected mutation sites were aligned with PharmVar listed *CYP2C9* allele table to identify the allelic variants. For novel variants not included in the allele table, bi-directional sanger sequencing were used for sequence verification with our recently published primers (25).

### 2.4 Expression of CYP2C9 variants in the yeast cells

Full-length cDNA of the typical defective *CYP2C9* allele (CYP2C9*3) was constructed using previously described overlap extension PCR amplification method (21). Similarly, cDNA of newly discovered variants were obtained with primer pairs listed in **Table 2** using wild type CYP2C9*1 cDNA as the PCR template. The resulting full-length cDNA fragments were double digested with EcoRI and XhoI, and ligated to EcoRI/XhoI digested pESC-OR vector to get the dual expression yeast vector pESC-OR-CYP2C9. Using previously described method, all newly detected CYP2C9 variants were highly expressed with co-expressed CYPOR enzyme in yeast cell microsomes (18). The quantification of expressed CYP2C9 proteins was performed according to our previously reported method (20).

**Table 2:** Primers used for the yeast expression vector construction.

Primer	*Sequence (5’-3’)	Amplicon	Variant	Note
2C9-UF	CCGA *GAATTC* ATGGATTCTCTTGTGGTCC	1499	wild type	EcoRI site listed in underline
2C9-UR	AACC *CTCGAG* TTAGACAGGAATGAAGCACAG			XhoI site listed in underline
2C9-L71R-F	ATTTTGGCCgGAAACCCA	1281	L71R	xx-R paired with UF
2C9-L71R-R	TGGGTTTCcGGCCAAAAT	236		xx-F paired with UR
2C9-P163S-F	AGGCCTCAtCCTGTGAT	1005	P163S	xx-R paired with UF
2C9-P163S-R	ATCACAGGaTGAGGCCT	511		xx-F paired with UR
2C9-T301M-F	GACAGAGAtGACAAGCA	590	T301M	xx-R paired with UF
2C9-T301M-R	TGCTTGTCaTCTCTGTC	926		xx-F paired with UR
2C9-E326K-F	CCAGGAAaAGATTGAACG	515	E326K	xx-R paired with UF
2C9-E326K-R	CGTTCAATCTtTTCCTGG	1002		xx-F paired with UR
2C9-C372R-F	CAGTGACCcGTGACATT	378	C372R	xx-R paired with UF
2C9-C372R-R	AATGTCACgGGTCACTG	1138		xx-F paired with UR
2C9-I389V-F	CCATATTAgTTTCCCTG	327	I389V	xx-R paired with UF
2C9-I389V-R	CAGGGAAAcTAATATGG	1189		xx-F paired with UR
2C9-H396Y-F	CTGTGCTAtATGACAAC	306	H396Y	xx-R paired with UF
2C9-H396Y-R	GTTGTCATaTAGCACAG	1210		xx-F paired with UR
2C9-N398H-F	GCTACATGACcACAAAG	302	N398H	xx-R paired with UF
2C9-N398H-R	CTTTGTgGTCATGTAGC	1214		xx-F paired with UR
2C9-G431R-F	TCTCAGCAaGAAAACGG	201	G431R	xx-R paired with UF
2C9-G431R-R	CCGTTTTCtTGCTGAGA	1315		xx-F paired with UR
2C9-I488F-R	AACCCTCGAGTTAGACAGGAAaGAAGC	1499	I488F	paired with UF

*The mutated site of each variant is illustrated as lower-case letter.

### 2.5 Enzymatic activity analysis

Based on our previously described methods (18, 22, 26), the drug metabolic activities of the wild type, typical defective variant CYP2C9.3, and seven allelic CYP2C9 variants found in this study were assessed with 2 typical CYP2C9 substrates: losartan and glimepiride. Briefly, reaction mixture contained 2-3 pmol of P450 from yeast microsomes, 5 μL purified cytochrome b5, and 2 μL substrates stock solution (dissolved in methanol) in 0.1M K_3_PO_4_ buffer (pH 7.5). The ultimate concentrations of losartan and glimepiride were 0.5-50 μM and 0.1-20 μM per reaction, respectively. After a 5 min pre-incubation, an NADPH-regenerating system (1.3 mM NADP+, 3.3 mM glucose-6-phosphate, 3.3 mM MgCl_2_, and 0.4 unit/mL glucose-6-phosphate dehydrogenase) was added to start the reaction at 37°C in a final volume of 200 μL and proceeded for 30 min (losartan) or 50 min (glimepiride). The incubation was terminated by adding an equal volume of the stop solution containing 150 μL acetonitrile and 50 μL internal standard midazolam (500 ng/mL). After vortexing, the incubated mixture was centrifuged at 12,000 × *g* for 5 min, and 200 μL aliquots were then removed and used for the following measurements. The incubations were performed in triplicate, and the mean values and S.D. from three experiments were provided for analysis.

Detection and quantification of the metabolites after incubation were performed on the ACQUITY UPLC I-Class/Xevo TQD IVD System (Waters, Milford, MA, USA). Aliquots of samples were placed into an ACQUITY UPLC BEH C18 column (2.1 mm × 50 mm; 1.7 μM; Waters), and the column temperature was maintained at 40°C. The initial mobile phase comprised A (pure acetonitrile, >98%) and B (ultrapure water), and the flow rate was 0.4 mL/min. The detection was performed on a triple quadrupole tandem mass spectrometer equipped with positive electrospray ionization (ESI) by multiple reactions monitoring (MRM) of the transitions. The linearity gradient elution condition for losartan was as following: 0–0.5 min (30%A), 0.5–1.0 min (30-95%A), 1.0–2.0 min (95%A), 2.0–2.3 min (95–30%A); The linearity gradient elution condition for glimepiride was set as 0–0.3 min (10-25%A), 0.3–2.0 min (25-95%A), 2.0–2.5 min (95%A), 2.5–2.6 min (95–10%A). The running time for all detections was 3.0 min. MRM transitions were m/z 437.20 → 235.00, m/z 507.30 → 126.10, and m/z 325.98 → 291.07 for E-3174, hydroxyglimepiride and midazolam, respectively. Nitrogen was used as the desolvation gas (1000 L/h) and cone gas (50 L/h). The dwell time was 0.063 s for E-3174 and 0.108 s for hydroxyglimepiride, the capillary voltage was set as 3.00 kV and the desolvation temperature was maintained at 500°C.

The enzymatic kinetic parameters Km, Vmax, and clearance rate Clint (Vmax/Km) were calculated by GraphPad Prism (version 9; GraphPad Software, Inc., CA, USA). IBM SPSS software (version 25.0, Magneto, New York, USA) was then used to evaluate the catalytic activity difference between the wild type and expressed variants by independent-samples T test.

## 3 Results

### 3.1 Distribution pattern of *CYP2C9* alleles in the Chinese Han population

As illustrated in **Figure 1**, two rounds of PCR amplification were included in the newly developed multiplex PCR amplicon sequencing method. All 9 exons and exon-intron regions of *CYP2C9* gene could be efficiently and specifically amplified after the first round PCR with multiplex PCR primers listed in **Table 1**. The products were pooled and purified with magnetic kit. Then, the purified amplicons were used for the second round of PCR amplification with universal primers to obtain the library for the second-generation sequencing. Using this system, we efficiently identified 39 allelic variants of *CYP2C9* in 1163 individuals, which include 16 previously reported nonsynonymous variations, 13 synonymous variations and 10 new nonsynonymous variations (**Table 3**). Similar to other studies, the most common defective allele in Chinese Han population is CYP2C9*3 with a allele frequency of 3.998% and 7.57% of studied subjects are heterozygote carrying *1/*3. In addition, 16 previously reported alleles (CYP2C9*2, *8, *13, *16, *29, *31, *34, *36, *37, *39, *45, *48, *53, *56, *60, and *75) were also detected in this study, and most of these allelic variants are heterozygous with the wild type with a total genotype frequency less than 4% which indicates that these alleles are rare in the Han Chinese populations (**Table 4**).

**Table 3:** Allelic *CYP2C9* variants identified in 1163 Chinese Han individuals.

Allele	Gene position	Nucleotide change	rsID	Amino-acid effect	Region	n	Allele frequency(%)
*36	1A>G	1A>G	rs114071557	SCM	exon 1	2	0.086
/	54A>T	54A>T	rs544425883	S18S	exon 1	3	0.129
*37	146A>G	146A>G	rs564813580	D49G	exon 1	4	0.172
new	3219T>G	212T>G	rs538852786	L71R	exon2	1	0.043
/	3235G>A	228G>A	rs17847036	V76V	exon2	12	0.516
*13	3276T>C	269T>C	rs72558187	L90P	exon2	8	0.344
*39	3300G>T	293G>T	rs762239445	G98V	exon2	3	0.129
/	3547C>A	369C>A		I123I	exon3	1	0.043
*45	3572C>T	394C>T	rs199523631	R132W	exon3	1	0.043
*2	3608C>T	430C>T	rs1799853	R144C	exon3	5	0.215
*8	3627G>A	449G>A	rs7900194	R150H	exon3	1	0.043
/	9098C>T	483C>T		A161A	exon4	2	0.086
new	9102C>T	487C>T		P163S	exon4	1	0.043
/	9155C>T	540C>T		S180S	exon4	1	0.043
*48	9235T>C	620T>C	rs1326630788	I207T	exon4	1	0.043
/	9245C>T	630C>T	rs773479415	S210S	exon4	1	0.043
/	10521A>G	738A>G		E246E	exon5	3	0.129
/	10533A>G	750A>G		E250E	exon5	1	0.043
/	10545A>G	762A>G		S254S	exon5	30	1.290
*29	33437C>A	835C>A	rs182132442	P279T	exon6	8	0.344
*16	33497A>G	895A>G	rs72558192	T299A	exon6	2	0.086
new	33504C>T	902C>T	rs757970831	T301M	exon6	1	0.043
*53	33551C>T	949C>T	rs1237225311	P317S	exon6	1	0.043
new	42515G>A	976G>A		E326K	exon7	1	0.043
*31	42519T>C	980T>C	rs57505750	I327T	exon7	2	0.086
*34	42543G>A	1004G>A	rs367826293	R335Q	exon7	1	0.043
*3	42614A>C	1075A>C	rs1057910	I359L	exon7	93	3.998
new	42653T>C	1114T>C		C372R	exon7	1	0.043
/	42676T>C	1137T>C	rs141283168	Y379Y	exon7	15	0.645
*56	47360A>G	1159A>G	rs764211126	I387V	exon8	1	0.043
new	47366A>G	1165A>G		I389V	exon8	1	0.043
new	47382C>T	1186C>T		H396Y	exon8	1	0.043
new	47393A>C	1192A>C		N398H	exon8	2	0.086
/	47398A>G	1197A>G		K399K	exon8	1	0.043
*75	47454A>C	1253A>C	rs1254213342	N418T	exon8	1	0.043
new	50164G>A	1291G>A		G431R	exon8	1	0.043
*60	50273T>C	1400T>C	rs767284820	L467P	exon9	1	0.043
/	50277C>T	1404C>T		D468D	exon9	1	0.043
/	50298A>T	1425A>T	rs1057911	G475G	exon9	92	3.955
new	50335A>T	1462A>T	rs1442749761	I488F	exon9	1	0.043

**Table 4:** Genotype frequencies of CYP2C9 allelic variants in 1163 Chinese Han individuals.

Genotype	n	Frequency (%)
*1/*1	1020	87.70
*1/*2	5	0.43
*1/*3	88	7.57
*3/*3	2	0.17
*3/*13	1	0.09
*1/*8	1	0.09
*1/*13	7	0.60
*1/*16	2	0.17
*1/*29	8	0.69
*1/*31	2	0.17
*1/*34	1	0.09
*1/*36	2	0.17
*1/*37	4	0.34
*1/*39	3	0.26
*1/*45	1	0.09
*1/*48	1	0.09
*1/*53	1	0.09
*1/*56	1	0.09
*1/*60	1	0.09
*1/*75	1	0.09
*1/*84	1	0.09

### 3.2 Identification of 10 new *CYP2C9* allelic variants

**Figure 2:**
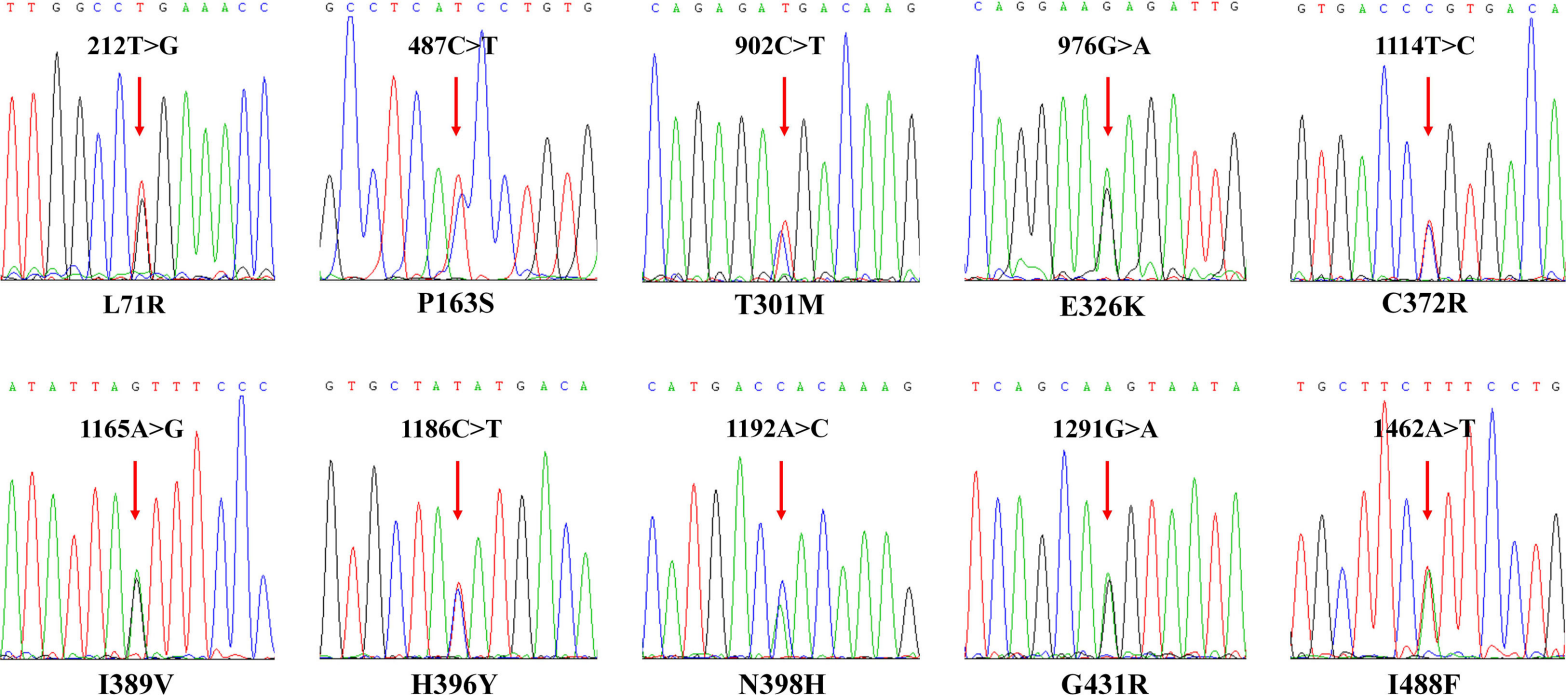
Variation verification of newly detected CYP2C9 variants. Sanger sequencing electropherogram pictures of newly detected CYP2C9 variants. The red arrow indicates that the variation sites detected in carriers and the amino acid substitutions are illustrated at the bottom of captured pictures.

In this study, 10 non-synonymous *CYP2C9* variations (L71R, P163S, T301M, E326K, C372R, I389V, H396Y, N398H, G431R, and I488F) were newly identified, which have not yet been nominated by the Pharmacogene Variation (PharmVar) Consortium (https://www.pharmvar.org/gene/CYP2C9). Their sequencing electropherogram pictures are shown in **Figure 2**. As illustrated in **Table 3**, these newly detected variants are located at almost all exons which include exon 2, exon 4, exon 6 - exon 9. Individuals carrying these variants were all heterozygous with wild type CYP2C9*1 and most of the variants could be detected in only one person, except for Asn398His which was found to be carried by two subjects. Specially, 7 of these 10 variants were reported for the first time and could be regarded as novel *CYP2C9* variants because they have not been registered by the dbSNP database or any other public databases currently.

### 3.3 Expression of newly detected CYP2C9 variants in yeast cells

**Figure 3:**
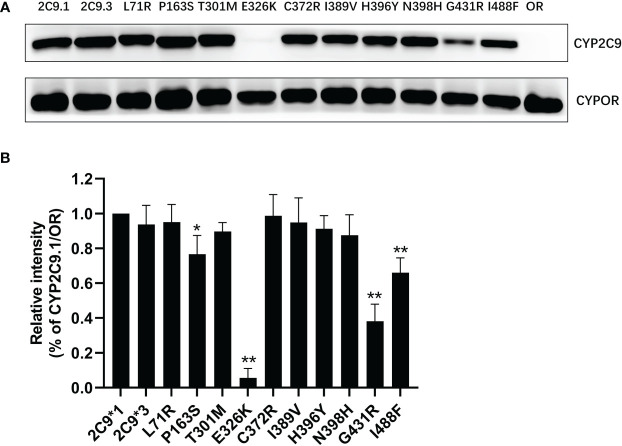
The immunoblotting results for expressed CYP2C9 variants in yeast microsomes. **(A)** Expressed CYP2C9 variants and CYPOR enzyme were detected by their corresponding antibodies after the SDS-PAGE gel separation. OR: microsome from yeast cells only expressing CYPOR enzyme. **(B)** Relative CYP2C9/OR intensities. Each bar represents the mean ± SD of three independently experiments. *P < 0.05, **P < 0.01 *vs* CYP2C9.1/OR.

In order to characterize the biological effects of newly detected CYP2C9 variants, the yeast expression system was used to efficiently co-express CYP2C9 enzyme and NADPH-cytochrome P450 oxidoreductase (CYPOR) according to the methods described previously (18). Immunoblot results indicated that most of newly detected CYP2C9 variants exhibited comparable protein expression level to that of wild type enzyme CYP2C9.1, except for variants Pro163Ser, Glu326Lys, Gly431Arg and Ile488Phe which showed obviously lower protein expression levels than the wild type (**Figure 3**).

### 3.4 Drug metabolic activity analysis of CYP2C9 variants

**Figure 4:**
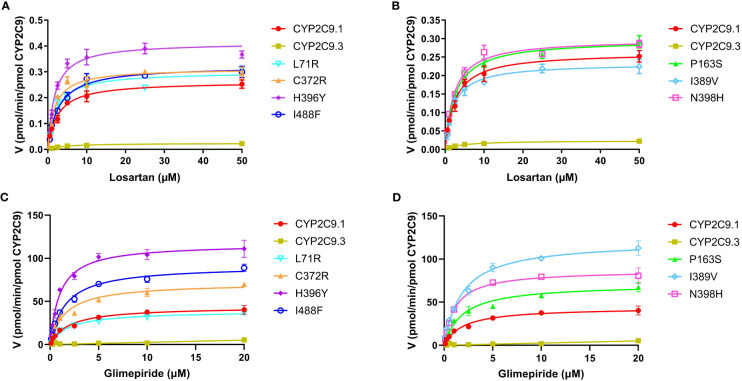
Michaelis-Menten curves of the enzymatic activities of expressed CYP2C9 variants toward losartan **(A, B)** and glimepiride **(C, D)**. Each point represents the mean ± S.D. of 3 separate experiments.

**Figure 5:**
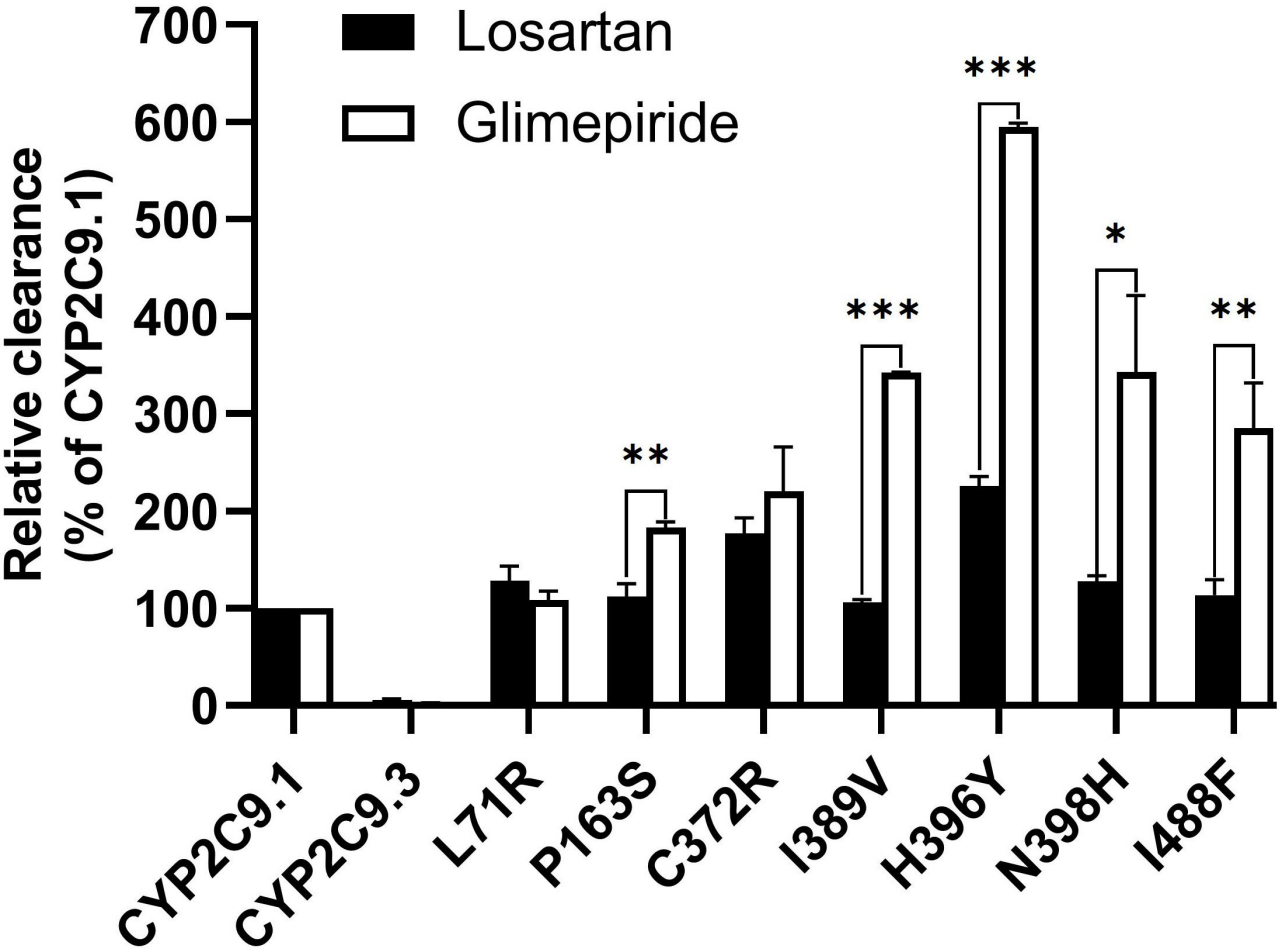
The relative clearance rates of losartan and glimepiride among wild type CYP2C9.1, typical defective variant CYP2C9.3 and 7 newly detected variants. *P < 0.05, **P < 0.01, and ***P < 0.005.

To better understand the impacts of newly detected CYP2C9 variants on drug metabolic activity of enzyme, two typical CYP2C9 mediated drugs losartan and glimepiride were included in this study. As a result, three variants (Thr301Met, Glu326Lys and Gly431Arg) showed no catalytic activities towards both drugs. Whereas 4 variants exhibited elevated activities for the metabolism of losartan (**Table 5**) and 6 variants exhibited increased intrinsic clearance rate for glimepiride, as compared with the wild type enzyme CYP2C9.1 (**Table 6**). These data indicated that most of newly detected CYP2C9 variants could significantly change the metabolic ability of enzyme (**Figures 4**, **5**).

**Table 5:** Enzyme kinetic values of recombinant wild type and CYP2C9 variants towards losartan.

Variants	Vmax (pmol/min/pmol P450)	Km (μM)	Clearance (Vmax/Km)	Relative clearance(/CYP2C9.1)
CYP2C9.1	0.26 ± 0.02	2.53 ± 0.24	0.10 ± 0.0079	100.00%
CYP2C9.3	0.02 ± 0.00*	4.35 ± 0.27*	0.01 ± 0.00053*	5.24%*
L71R	0.32 ± 0.02*	2.47 ± 0.59	0.13 ± 0.025	128.16%*
P163S	0.30 ± 0.01*	2.60 ± 0.25	0.11 ± 0.0092	109.16%
C372R	0.31 ± 0.00*	1.72 ± 0.09*	0.18 ± 0.0068*	174.31%*
I389V	0.23 ± 0.02	2.16 ± 0.36	0.11 ± 0.012	102.94%
H396Y	0.42 ± 0.01*	1.80 ± 0.26*	0.23 ± 0.026*	223.68%*
N398H	0.30 ± 0.01*	2.28 ± 0.20	0.13 ± 0.0093*	125.38%*
I488F	0.32 ± 0.02*	2.81 ± 0.53	0.12 ± 0.016	111.27%

Data are presented as the mean ± S.D. of 3 different expression experiments. *P < 0.05 vs. wild-type CYP2C9.1.

**Table 6:** Enzyme kinetic values of recombinant wild type and CYP2C9 variants towards glimepiride.

Variants	Vmax (pmol/min/pmol P450)	Km (μM)	Clearance (Vmax/Km)	Relative clearance(/CYP2C9.1)
CYP2C9.1	45.12 ± 6.04	2.25 ± 0.80	21.39 ± 5.77	100.00%
CYP2C9.3	12.70 ± 2.47*	29.30 ± 4.75	0.43 ± 0.014*	2.02%*
L71R	37.85 ± 1.44	1.69 ± 0.33	22.85 ± 4.042	108.45%
P163S	69.21 ± 2.49*	1.85 ± 0.45*	38.92 ± 9.57	182.72%*
C372R	72.28 ± 1.61*	1.60 ± 0.15	45.43 ± 4.65*	219.92%*
I389V	122.23 ± 7.17*	1.86 ± 0.34	66.89 ± 9.11*	319.66%*
H396Y	117.10 ± 5.56*	1.04 ± 0.11	113.68 ± 11.67*	546.32%*
N398H	89.29 ± 3.82*	1.13 ± 0.09	77.27 ± 2.73*	379.09%*
I488F	90.87 ± 5.32*	1.55 ± 0.23	59.18 ± 5.42*	284.93%*

Data are presented as the mean ± S.D. of 3 different expression experiments. *P < 0.05 vs. wild type CYP2C9.1.

## 4 Discussion

In this study, we developed a timesaving and cost-effective genotyping method for *CYP2C9* gene. This method is based on the combination of MPCR (Multiplex polymerase chain reaction) and NGS (Next generation sequencing) techniques and has many advantages over traditional Sanger sequencing method. Firstly, this method is easy to be operated in a large scale with automatic protocol, leading to the lower cost, reduced man-made errors and improved accuracy than Sanger method; Secondly, newly developed method can present overall genetic information of *CYP2C9* gene in the target region, that can be used for the genotyping of previously reported alleles and for the discovery of novel variants with unreported mutations, simultaneously; Finally, short time, typically only 2-3 weeks, is needed for the genotyping of hundreds of the samples. In contrast, for traditional Sanger sequencing method, several months maybe needed for the sequencing and analyzing of all 9 exons of *CYP2C9* in a large scale. In brief, our method not only reduces the costs for genotyping, but also greatly improves the efficiency and accuracy of sequencing, favoring its application in large sample scale, multi-center or multi-targets genotyping projects.

Like other *CYP2C* members, *CYP2C9* gene shows marked differences in the allelic frequency in different biogeographic groups and races. These genetic polymorphisms are highly related to the adverse drug reactions (ADRs), especially for the drugs with narrow therapeutic window (27), such as the hypoglycemia caused by hypoglycemic drugs (28), the gastrointestinal bleeding caused by non-steroidal anti-inflammatory drugs (29, 30), and severe bleeding caused by anticoagulation therapy (31, 32), etc. Therefore, digging out the “special subgroups” with abnormal drug metabolism in the population is one of the key factors for reducing the occurrence of ADRs in the clinic. For instance, warfarin is the most commonly used oral anticoagulant, but its therapeutic index is narrow and wildly variable among different patients. Genetic polymorphisms of *CYP2C9* and Vitamin K epoxide reductase complex subunit 1 *(VKORC1)* are one of the most concerned factors for the optimal warfarin dose determination in clinic (33). S-warfarin is mainly metabolized *via* CYP2C9 to 7-hydroxy warfarin. Typical missense variant CYP2C9*3 caused a remarkable decrease in the S-warfarin clearance rate, leading to the increased risk of venous thromboembolism and bleeding in patients (13). Our recent studies revealed that a lot of rare *CYP2C9* alleles are carried by Chinese individuals and most of missense mutations in *CYP2C9* gene are highly related to the low dose of warfarin in Chinese population (18, 20, 34). In this study, we developed one time-saving and cost-effective genotyping method for *CYP2C9* and performed a genetic screening in 1163 Chinese individuals. Similar to our previous study, CYP2C9*3 is the most prevalent defective alleles in Chinese population although the allele frequency detected in this study is slightly higher than previous report (17). Additionally, CYP2C9*13 and *29 exhibited relatively higher frequencies than other allelic variants which is in agreement to our previous reports (17, 25). For the first time, we reported one Chinese individual carrying allele CYP2C9*8 which was previously regarded as only limited to individuals of African ancestry (35). Specially, we detected 10 new allelic variants that have not been listed on the PharmVar consortium website (**Table 3**; **Figure 2**). These data indicated that *CYP2C9* was highly polymorphic in Chinese population and more attention should be paid to the distribution pattern and its potential clinical application in clinic, considering that more than 1.4 billion people lived in the mainland of China.

Glimepiride is one of the most used oral sulfonylureas (SU) drugs in the clinical treatment of type 2 diabetes mellitus (T2DM). Hypoglycemia is the most common adverse effect related to SU therapy and severe hypoglycemia might significantly increase the cost of medication and decrease the quality of life for T2DM patients (36, 37). Since CYP2C9 is the major enzyme involved in SUs metabolism, the risk of hypoglycemia induced by SUs would be elevated in deleterious CYP2C9 variant allele carriers (38). According to a recent meta-analysis, CYP2C9 variant alleles have increased risk of hypoglycemia than wild-type CYP2C9*1/*1 after the SUs treatment. The incidence of hypoglycemia would be increased by 80% in CYP2C9*2 carrier (39). Previous studies have also reported that the AUC of tolbutamide was increased by 150% and 190% in CYP2C9*1/*2 and *1/*3 carriers, respectively (40); Similarly, for glimepiride, the AUC was increased by 167% in CYP2C9*3 carriers in comparison to CYP2C9*1/*1 individuals (41). In this study, the *in vitro* metabolic activity analysis results revealed that 3 newly detected CYP2C9 variants had no catalytic activity for glimepiride metabolizing and carriers for these variants might exhibit significantly reduced drug metabolizing activity for SU. However, most of other newly detected allelic variants exhibited significantly increased enzyme activity for glimepiride metabolism which indicated that carriers with these variants might possess the higher drug metabolizing activity for SU than individuals with wild type CYP2C9*1/*1. (**Figures 4**, **5** and **Table 6**). These data indicated that different amino acid substitution at different sites of CYP2C9 protein had different effects on the drug metabolizing activity of enzyme.

Typical tertiary structure of a cytochrome P450 enzyme mainly consists of twelve α-helices (A-L) and four β-sheets (1–4) with the heme locating between the helices I and L. There are six substrate recognition sites (SRSs) in CYP2C9 enzyme which locate at the amino acids 96-117 (between the helices B and C), 198-205 (between the helices F and G), 233-240 (between the helices F and G), 286-304 (in the center of the helix I), 359-369 (at the N-terminus of β strand 1-4), and 470-477 (at the turn at the end of β sheet 4), respectively (42). In this study, 3 allelic variants, Thr301Met, Glu326Lys and Gly431Arg showed activity deficiency for both losartan and glimepiride. In the crystal structure of CYP2C9, Thr301 is involved in the SRS4, and Gly431 belongs to heme-binding motif residues. Therefore, the amino acid substitution at position 301 or 431 is estimated to affect the substrate recognition or heme propionate binding capacity for CYP2C9. Similar to our results, another allelic variant at position 301, Thr301Lys, also showed no enzymatic activity (43). These data indicated that Thr301 might be crucial for the drug metabolic activity of enzyme. Glu326 is located at the helix J of CYP2C9 and it has a strong binding strength with 5 amino acid residues within 5Å distance. Previous study revealed that variant Glu326Asp (CYP2C9*65) had deleterious effect in SIFT and Polyphen prediction (12). Combined with the data in this study, it is estimated that the replacement of Glu326 might influence the enzyme activity significantly. Different from these 3 defective variants, most of other newly detected variants exhibited significantly increased metabolizing activities towards both losartan and glimepiride *in vitro* (**Figure 5**; **Tables 5**, **6**). These data indicated that carriers with these allelic variants might have higher metabolizing activity for CYP2C9 mediated drugs.

In summary, we developed a time-saving next generation sequencing based method for *CYP2C9* genotyping and performed a large-scale polymorphic screening of *CYP2C9* gene in Chinese Han population. Totally 16 previously reported allelic variants and 10 new non-synonymous variations were detected in this study. When expressed in yeast microsomes, most of newly detected variations showed similar protein expression level to wild type. Further drug metabolic activity analysis revealed that 3 variants were loss of function isoforms and most of other newly detected variants exhibited significantly increased metabolizing activities for both losartan and glimepiride. Our study greatly enriched the knowledge of genetic polymorphism of *CYP2C9* in Chinese Han population, and the clinical significance of newly detected CYP2C9 alleles still needs further investigation by enlarging the sample size and deep correlation analysis between genetic information and clinical features.

## Data availability statement

The datasets presented in this study can be found in online repositories. The names of the repository/repositories and accession number(s) can be found below: Genbank-BankIt2667312 Seq_C487T OQ376733, BankIt2667312 Seq_G976A OQ376734, BankIt2667312 Seq_T1114C OQ376735, BankIt2667312 Seq_A1165G OQ376736, BankIt2667312 Seq_C1186T OQ376737, BankIt2667312 Seq_A1192C OQ376738, BankIt2667312 Seq_G1291A OQ376739.

## Ethics statement

The studies involving human participants were reviewed and approved by the ethics committee of Beijing Hospital. The patients/participants provided their written informed consent to participate in this study.

## Author contributions

DD, JY, and HC contributed to conception and design of the study. QiZ, YQ, SW, FZ, LZ, QuZ, PG, YH, and HY performed the experiments. QiZ, YQ, SW, FZ, QuZ, QL, JC, HW, and DW performed the statistical analysis. QiZ wrote the first draft of the manuscript. YQ and DD wrote sections of the manuscript. All authors contributed to manuscript revision, read, and approved the submitted version.
